# Homocysteine Metabolism in Children and Adolescents: Influence of Age on Plasma Biomarkers and Correspondent Genotype Interactions

**DOI:** 10.3390/nu11030646

**Published:** 2019-03-16

**Authors:** Helena Caldeira-Araújo, Ruben Ramos, Cristina Florindo, Isabel Rivera, Rita Castro, Isabel Tavares de Almeida

**Affiliations:** 1 Faculty of Life Sciences, University of Madeira, Campus da Penteada, 9000-390 Funchal, Portugal; helena.caldeira@staff.uma.pt; 2 Centro de Química da Madeira, University of Madeira, Campus da Penteada, 9000-390 Funchal, Portugal; 3 Metabolism and Genetics Laboratory, Research Institute for Medicines (i.Med.ULisboa), Faculty of Pharmacy, University of Lisbon, 1649-003 Lisbon, Portugal; ruben_ramos120@hotmail.com (R.R.); cristinaflorindo@ff.ulisboa.pt (C.F.); iarivera@ff.ul.pt (I.R.); italmeida@ff.ul.pt (I.T.d.A.); 4 Department of Biochemistry and Human Biology, Faculty of Pharmacy, University of Lisbon, 1649-003 Lisbon, Portugal; 5 Department of Nutritional Sciences, The Pennsylvania State University, University Park, PA 16802, USA

**Keywords:** vitamin B12, methylmalonic acid, folate, homocysteine, MTHFR

## Abstract

Background: Imbalance of homocysteine (Hcy) metabolism links with several pathologies; nevertheless, it is poorly characterized in pediatric populations. This study investigated the impact of age on plasma concentrations of Hcy and relevant biomarkers along with correspondent genotype interactions. Methods: A healthy pediatric cohort aged 9 (*n* = 195) and 17 (*n* = 128) years old (yo) was studied. Immunoassays and GC-MS-SIM-mode quantified plasma levels of Hcy and biomarkers. PCR-RFLP or quantitative-PCR assays assessed common variations in related genes. Results: Age impacted on levels of Hcy and metabolic markers: older children presented with the lowest folates and total-cobalamin (tCbl), while with the highest Hcy concentrations, whereas methylmalonic acid (MMA) and holotranscobalamin (Holo-TC) levels remained similar in 9-yo and 17-yo children. The relationships between B-vitamins and metabolic markers were also dependent on age. Only in the older children, MMA correlated with tCbl and Holo-TC, and MMA levels were markedly higher in the 17-yo subjects presenting with the lowest quartiles of Holo-TC concentrations. Lastly, age also impacted on the correlations between genotype and biomarkers. In the 17-yo group, however not in the 9-yo children, tHcy differed between *MTHFR* 677 genotypes, with subjects who had the *MTHFR* 677TT genotype displaying the highest tHcy concentrations. Conclusions: Age impacts on the Hcy metabolism dynamics in a pediatric population.

## 1. Introduction

The homocysteine (Hcy) metabolism is a metabolic network centered on the folate and methionine cycles in which one-carbon (1-C) groups are transferred, supporting multiple physiological processes, including nucleotide biosynthesis, amino acid homeostasis, epigenetic maintenance and redox defense [1,2]. Consequently, a disturbed Hcy metabolism is associated with common pathologies, such as neurodegenerative and cardiovascular (CVD) diseases and cancer [3,4,5,6,7,8,9,10,11].

The metabolisms of Hcy, folate and vitamin B_12_ (or cobalamin, Cbl) are biochemically linked sharing several metabolic intermediates [9]. Hcy is formed from the essential amino acid methionine. Once formed, Hcy may be conserved and remethylated back to methionine by methionine synthase (MS). This enzyme requires the presence of methylcobalamin (methylCbl), as a cofactor, to carry on its function. In turn, the synthesis of methylCbl needs the intervention of 5-methyltetrahydrofolate (5-methyl-THF), the main circulating form of folate [7]. Then, 5-methyl-THF transfers the methyl group to Cbl, thus releasing tetrahydrofolate (THF) that will sustain several 1-C transfer reactions. Transcobalamin II (TC), a plasma protein, binds Cbl and transports it to cells. Transcobalamin-bound vitamin B_12_ is designated as holotranscobalamin (Holo-TC) [12]. The common term total Cbl (tCbl) also includes other circulating forms of Cbl, of which its functions are unclear. However only the Holo-TC fraction enters into the cells and releases the Cbl [12]. Consequently, insufficient levels of methylCbl may be associated with a conditional folate deficiency [7,13], impairing the remethylation of Hcy, which will accumulate in plasma [7,9,13,14], reflecting the insufficient Cbl and folate intracellular status. Moreover, Cbl is also converted to adenosylCbl, which is used for the metabolism of methylmalonyl-CoA [15,16]. Therefore, an insufficient B_12_/Cbl cell status leads to the build-up of plasma Hcy and methylmalonic acid (MMA), formed from methylmalonyl-CoA [17,18,19,20]. A more comprehensive description of the Hcy metabolism can be found in references [8,9,10].

Genetic background may also impact on the levels of plasma Hcy. Gene variants coding for enzymes involved in Hcy metabolism may modulate the correspondent enzyme activities [2]. MTHFR participates in the remethylation of Hcy to methionine. The *MTHFR* 677C>T polymorphism (p.A222V) results in a thermolabile variant of MTHFR with a decreased enzyme activity [21,22,23,24] and is a well-established genetic determinant of elevated plasma tHcy (total homocysteine; all the circulating forms of Hcy) levels [25,26,27]. Other common gene variations, which are also involved in the Hcy remethylation, have been reported, albeit with less consistent effects on Hcy circulating levels [2,27,28]. These include the *MTHFR* 1298A>C (p.E429A) [29] and the *MTR* 2756A>G (p.D919G) [30] and *MTRR* 66A>G, the last two being related with MS activity [31]. Further, variations in the TC encoding gene, or *TCN2*, may also affect the circulating concentrations of tHcy. The *TCN2* 776C>G (p.P259R) and 67A>G (p.I23V) variants diminish the TC’s ability to bind and transport cobalamin to tissues, causing an accumulation of Hcy due to deficient remethylation [32,33]. 

Despite the pivotal importance of Hcy metabolism for cellular homeostasis, data are scarce in the pediatric population, which is particularly vulnerable due to the higher requirements of nutrients for healthy growth and development. Adequate reference values for Hcy and related metabolic biomarkers in the pediatric population are important in clinical decision-making, with possible health impacts. Nevertheless, providing reference values in school-age children is challenging, and most of the available reference values for Hcy are obtained from adults. Another question in establishing pediatric reference values is to consider the potential effect of specific ages on Hcy metabolism. For instance, differences in physical size, organ maturity, fluid body compartments, immune and hormone responsiveness are likely to affect the concentrations of Hcy in children and youth. Here, we investigated the Hcy metabolism in 9 and 17 year-old (yo) subjects by exploring the impact of age, gender and genotype on the levels of tHcy, MMA and B-vitamins.

## 2. Materials and Methods

### 2.1. Subjects

Two groups of a pediatric population from Madeira Island, Portugal: 128 adolescents aged 17-yo (71 females, 57 males) and 195 children aged 9-yo (88 females, 107 males) were studied. Subjects were enrolled from schools that were selected from different areas of the island. All participants were Caucasian. Data on dietary and lifestyle habits were collected and used as a basis for the selection of eligible individuals. All subjects were under a Mediterranean diet and the intake of protein and vegetables/fruit followed the recommended dose/age. Dietary intake was evaluated by application of a semi-quantitative Food Frequency Questionnaire. Acute and chronic illnesses were the exclusion criteria. All selected individuals presented an irrelevant anamnesis and did not take drugs and vitamin supplementation. For all participants, written informed consents were obtained. All procedures were conducted in accordance with the International Ethical Guidelines for Biomedical Research Involving Human Subjects and the study was approved by the Scientific and Research Committee of our Institute at Madeira University. 

### 2.2. Anthropometric Data

All participants were assessed for Body Mass Index (BMI), waist circumference, systolic and diastolic pressure using standard methods.

### 2.3. Blood Sample Collection and Biochemical Analyses

Overnight fasting blood samples were collected by venipuncture in EDTA containing tubes. Part of the blood was used for DNA extraction (as described below) and the remaining blood was immediately centrifuged at 4000 *g* for 10 min at 4 °C. Plasma was collected, divided into several aliquots and stored at −80 °C until analysis.

Plasma levels of total cholesterol, HDL-cholesterol, LDL-cholesterol, triacylglycerols, creatinine and uric acid were quantified by standard routine methods (Unicel DXC, Beckman Coulter Inc., Brea, CA, USA).

Plasma tHcy and folate were evaluated, respectively, by a fluorescence polarization immunoassay and an ion-capture enzyme immunoassay from IMx Systems (Abbott Laboratories, Chicago, IL, USA), according to the manufacturer’s instructions. Plasma tCbl and Holo-TC were determined by a microparticle enzyme immunoassay using an AxSYM analyser (Abbott Laboratories, Chicago, IL, USA). Plasma MMA concentration was determined by gas chromatography coupled with mass spectrometry in a selected ion mode (GC-MS-SIM) following a laboratory procedure adapted from a previously published method [34]. 

### 2.4. Genotype Analyses 

Genomic DNA was isolated from peripheral blood leucocytes (Puregene, DNA purification system, Gentra Systems) and was stored at 4 °C until use. Identification of the allelic variants *MTHFR* 677C>T, *MTHFR* 1298A>C, *MTR* 2756A>G, *TCN2* 67A>G and *TCN2* 776C>G was performed by PCR-RFLP assay using *Hinf*I, *Mbo*II, *HaeIII*, *RsaI* and *ScrFI* endonucleases, respectively [16,30,35]. After restriction enzyme digestion, PCR products were evaluated by gel electrophoresis analysis. Allelic discrimination of *MTRR* 66A>G variants was performed by real-time PCR (iQ5 Real-Time PCR Thermocycler, BioRad, California, USA) using TaqMan probes (TaqMan^®^ SNP Genotyping Assays, Applied Biosystems, Waltham, MA, USA).

### 2.5. Statistical Analysis

All statistical analyses were performed using the program IBM SPSS Statistics 22.0. Normality of data and homogeneity of variances were evaluated, respectively, by *Kolmogorov-Smirnov* and *Levene* tests. Data were expressed as the mean ± standard deviation (SD). Continuous variables were compared by independent samples T-test and by one-way analysis of variance (ANOVA). Spearman correlation coefficients were calculated to determine the relationship between all metabolites and vitamins. A *p*-value lower than <0.05 was considered statistically significant. 

## 3. Results

### 3.1. Anthropometric and Biochemical Parameters 

Anthropometric and routine biochemical parameters (data not shown) of the studied population were stratified according to age and gender. All the analyzed parameters were within the normal range according to age and gender. Between genders, significant differences were observed in the 9-yo group only for the systolic pressure (*p* < 0.05), which was higher in females. In the 17-yo group, males presented significantly higher systolic pressure (*p* < 0.01), waist circumference (*p* < 0.001) and creatinine (*p* < 0.001), however lower HDL-cholesterol (*p* = 0.001) than females. All the included individuals were eutrophic according to BMI cut-off values of the World Health Organization (WHO) Growth Reference Data 2007. 

### 3.2. Age and Plasma Biomarkers

**Figure 1:**
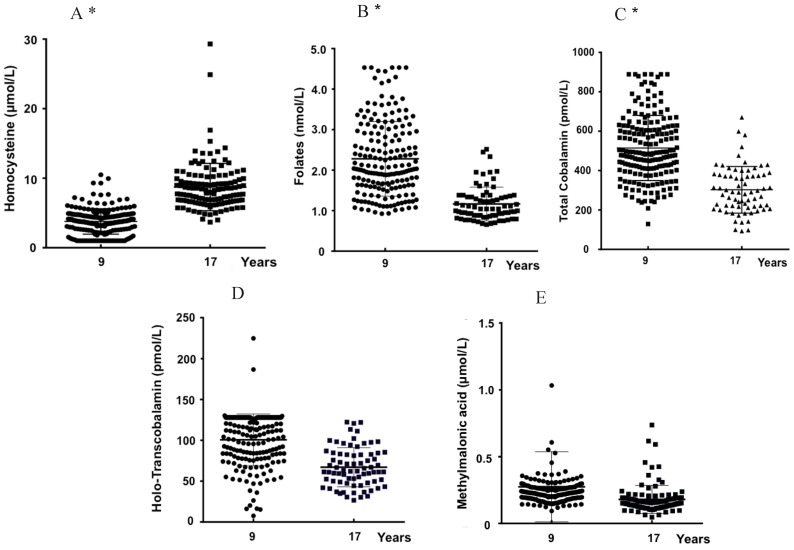
Distribution of the concentrations of total homocysteine (**A**), folates (**B**), cobalamin (**C**), holo-transcobalamin (**D**) and methylmalonic acid (**E**) for the 9-yo (*n* = 195) and 17-yo (*n* = 128) children. * *p* < 0.05, Student *T* test 9-yo versus 17-yo.

The levels (mean ± SD) of plasma biomarkers according to age and gender are displayed in Table 1. Figure 1A–E represents the distribution of the same biomarkers in the 9- (*n* = 195) and 17-yo (*n* = 128) individuals.

Concerning the putative influence of gender within each age-group (Table 1), no significant differences were observed except for tHcy concentrations, which were significantly higher in 17-yo males (Hcy: 10.2 ± 4.0 µM) than in same-age females (Hcy: 7.5 ± 2.2 µM). When comparing the 17-yo subjects with the 9-yo individuals within the same gender, plasma concentrations of tHcy (µM) increased significantly with age, while those of folate, tCbl and Holo-TC decreased significantly. Moreover, MMA levels decreased significantly with age in females, however not in males.

When both genders were considered together (Figure 1), plasma concentrations of tHcy (µM) increased significantly (3.8 ± 1.8 to 8.7 ± 3.4; *p* < 0.001) with age, while those of folate (nM) (22.8 ± 9.3 to 11.6 ± 4.2; *p* < 0.001) and tCbl (pM) (302.5 ± 118.3 to 286.5 ± 97.2; *p* < 0.001) decreased significantly. No significant differences were observed for Holo-TC and MMA plasma levels.

Taking all subjects into account, as expected, tHcy showed a significant negative linear association with folate, Holo-TC and tCbl (Spearman correlation coefficient, *r* = −0.625; −0.380 and −0.519, respectively, *p* < 0.01). Moreover, a positive linear association was observed between Holo-TC and tCbl levels (*r* = 0.426, *p* < 0.01). 

**Figure 2:**
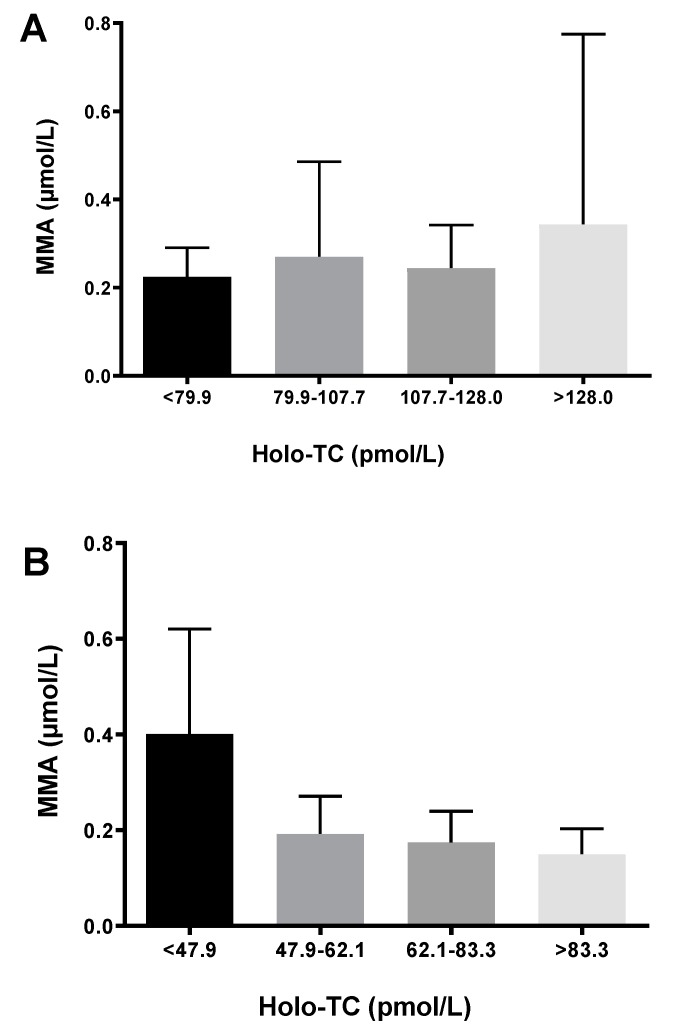
Methylmalonic acid (MMA) concentrations by quartiles of holo-transcobalamin (Holo-TC) in the 9-yo (**A**) and 17-yo (**B**) children.

Plasma MMA levels were significantly correlated with tCbl (*r* = −0.335, *p* < 0.05) and with Holo-TC (*r* = −0.515, *p* < 0.01) in the 17-yo subjects, however not in the 9-yo group. Moreover, MMA concentrations for each age group were plotted according to Holo-TC quartiles (Figure 2), and only in the 17-yo group, the highest MMA levels were associated with the lowest Holo-TC levels. Taken together, these observations confirm MMA as a good predictive metabolic marker of cobalamin status in the older children, however also suggest that MMA concentrations do not reflect the cobalamin status in the younger children. 

### 3.3. Genotypes, Age, and Biomarkers

Genotype and allele frequencies for the whole group are displayed in Table 2, with all genotype frequencies consistent with the Hardy-Weinberg equilibrium.

The plasma concentrations of vitamins and metabolic markers were stratified according to the various single nucleotide polymorphisms (SNPs), and the results are summarized in Table 3 (9-yo group) and 4 (17-yo group). In the 9-yo group, no significant differences were observed for any of the evaluated biomarkers among the different genotypes. However, in the 17-yo group, significant differences (*p* < 0.05) were observed for tHcy levels among the *MTHFR* 677 genotypes and tCbl concentrations among *MTR* 2756 genotypes.

## 4. Discussion

Disruption of Hcy metabolism leads to hyperhomocysteinemia, which has been associated with different pathological conditions, including vascular and neurodegenerative diseases [11,36,37,38]. The causes of hyperhomocysteinemia are multifactorial and include insufficient levels of the B-vitamins, particularly folates and Cbl, which are cofactors and co-substrates in Hcy metabolism [36,37,39]. European dietary surveys disclosed a widespread prevalence of suboptimal plasma concentrations of folates and tCbl in various age groups, despite apparent adequate intakes [37,39,40]. This fact is of particular concern in young age groups in which it has been related to developmental delay and irreversible neurological damage [41,42,43,44,45]. Nevertheless, few studies have examined the Hcy metabolism in pediatric populations. 

This study was conducted in a healthy pediatric population of children (aged 9-yo) and adolescents (aged 17-yo). The impact of age on Hcy-related metabolism biomarkers and gender differences were evaluated along with that of common variants in genes (*MTHFR*, *MTR* and *MTRR*) encoding enzymes involved in the folate/Cbl dependent Hcy remethylation cycle, or in the *TCN2* gene, which encodes the protein that delivers Cbl to cells. 

The results showed an increase of tHcy concentration and a decrease of folate and Cbl levels with age (Figure 1 and Table 1). Moreover, gender differences were found, however only in the 17-yo group, for tHcy level, with a higher mean value in males. These observations concur with previously published data [46,47,48,49]. The plasma tHcy values are influenced not only by environmental and genetic factors, however also by nutritional ones, including different fortification policies worldwide [47,48]. Therefore, differences between the dietary habits and lifestyle between the younger children and adolescents may explain the observed decrease of folate and Cbl levels, which harm Hcy metabolism, contributing to Hcy accumulation. The increase in tHcy concentrations is also influenced not only by hormonal factors, which support gender differences, however also by the increment of body mass, along with healthy development, which demands a high ratio of creatine synthesis. This metabolic reaction is the leading consumer of the methyl groups donated by S-adenosylmethionine, which is formed during the conversion of methionine to Hcy [9].

The 9-yo group displays a tHcy plasma mean level (3.8 µmol/L) (Table 1) that is lower than those reported for Dutch children aged 6–10 yo (6.2 µmol/L) [49]; for Belgian children aged 5–9 and 10–14 yo (6.2 to 7.1 µmol/L, respectively) [46]; and for American children aged 6–11 yo (5.0 to 5.4 µmol/L) [48]. The observed difference may be attributable to the Mediterranean diet, characterized by a high consumption of fresh vegetables and fruits, which, at this age, is still under family control, therefore contributing to the high levels of plasma vitamins observed and its lowering effect on tHcy concentrations.

The isomerization of L-methylmalonyl coenzyme A by methylmalonyl coenzyme A mutase, using AdoCbl as a cofactor, and its independence from folate metabolism, have made MMA an attractive biomarker of Cbl deficiency [41]. In this study, despite a decrease in tCbl and Holo-Cbl levels with age, MMA plasma concentrations in the 9- and 17-yo groups were not significantly different (Figure 1 and Table 1). This observation agrees with the NHANES study, which states that circulating MMA is generally stable at an age below 20 years and only increases later, with an upward trend after 40 years of age [50]. In the present report, MMA levels did not correlate with tCbl or with Holo-TC levels in the 9-yo group, while significant correlations were observed in the adolescents’ group. This observation suggests that, in pediatric populations, the usefulness of MMA as a metabolic biomarker of cobalamin status needs to be further investigated. It should be taken into account that several factors may cause variability of plasma MMA, among them alterations in the gut microbiota [51], as well as in the metabolism of odd-chain fatty acids and amino acids precursors of MMA (such as methionine, isoleucine and threonine) [18,20,41,50]. Moreover, genetic background may also influence plasma MMA concentrations. Recently, in an Irish population, a common polymorphism in the *HIBCH* gene, encoding 3-hydroxyisobutyryl-CoA hydrolase, was strongly associated with elevated MMA concentrations independently of tCbl or Holo-TC levels [52]. 

The frequency of the *MTHFR* 677TT genotype was 9.2% (Table 2), similar to other European populations, including the Netherlands (8.2%) [49], Germany (9.7%) [25] and Czech Republic (10%) [53,54], however was slightly lower than the one reported for Northern Ireland (13.5%) [55], France (11.8%) and Spain (11.8%) [56]. Moreover, *MTHFR* 1298CC, *MTR* 2756GG, *MTRR* 66GG, *TCN2* 776GG and *TCN2* 67GG genotype frequencies were also comparable to those reported for other Caucasian European populations [53,54,55,57].

*MTHFR* 677C>T is a well-established genetic determinant of elevated plasma tHcy [25,26,27], and lower folate concentrations significantly enhance this effect. Accordingly, in the 9-yo group (Table 3), which presents high plasma folate levels, tHcy plasma concentrations did not differ significantly among the *MTHFR* 677 genotypes, suggesting that folate levels modulate the expected correlations between genotype and metabolic markers. Supporting that correlation, in the 17-yo group (Table 4), subjects bearing the *MTHFR* 677TT genotype displayed significantly higher tHcy concentrations than those bearing the wild-type genotype. A similar situation was observed concerning the *MTR2756GG* genotype, since it was associated with significantly decreased tCbl plasma levels in 17-yo adolescents (Table 4). 

## 5. Conclusions

Most available reference values for Hcy plasma concentrations have been established in adults. Therefore, the present study adds new information reporting plasma concentrations of tCbl, Holo-Cbl, tHcy and MMA in two groups, 9-yo and 17-yo, of a healthy pediatric cohort. The study of genetic variants related to Hcy metabolism in young pediatric populations may also allow for better knowledge of native phenotypes, as time was not enough for environmental factors to modify them substantially.

In conclusion, this work contributes to a better characterization of Hcy metabolism in pediatric populations. Moreover, it reinforces the notion that regarding the plasma concentrations of relevant biomarkers and their interactions with genotype, age powerfully impacts on Hcy metabolism within these populations. Extensive studies are needed to assess further knowledge concerning the impact of age on the modulation of Hcy metabolism and related pathologies, enabling the implementation of disease prevention measures.

## Figures and Tables

**Table 1:** Plasma concentrations (mean ± SD) of vitamins and metabolic markers in the studied population, stratified according to gender and age.

	Males					Females					*p ^ϕ^*	*p ^Ψ^*
	*N*	9-Year-Old	*N*	17-Year-Old	*p* ^1^	*N*	9-Years-Old	N	17-Year-Old	*p* ^2^		
**tHcy (µM)**	107	3.8 ± 1.8	52	10.2 ± 4.0	<0.001	88	3.8 ± 1.9	67	7.5 ± 2.2	<0.001	0.811	<0.001
**Folates (nM)**	96	22.9 ± 9.1	39	11.1 ± 3.5	<0.001	80	22.8 ± 9.5	37	12.2 ± 4.7	<0.001	0.969	0.229
**tCbl (pM)**	104	502.1 ± 163.1	35	286.5 ± 97.2	<0.001	83	530.6 ± 168.2	36	318.0 ± 135.3	<0.001	0.243	0.265
**Holo-TC (pM)**	99	103.2 ± 28.5	38	69.9 ± 25	<0.001	80	97.4 ± 35.1	37	62.4 ± 24.8	<0.001	0.226	0.196
**MMA (µM)**	99	0.30 ± 0.31	48	0.21 ± 0.14	0.082	85	0.25 ± 0.19	62	0.18 ± 0.16	<0.001	0.211	0.087

^1^ Independent samples *T*-test between age-groups within males; ^2^ Independent samples *T*-test between age-groups within females. *ϕ* Independent samples *T*-test between 9-yo males and females; *Ψ* Independent samples *T*-test between 17-yo males and females. Total Homocysteine (tHcy); total cobalamin (tCbl); holo-transcobalamin (Holo-TC); methylmalonic acid (MMA).

**Table 2:** Genotypic and allelic frequencies of single nucleotide polymorphisms (SNPs) in *MTHFR, TCNII, MTR* and *MTRR* genes in the whole studied group (*n* = 323).

SNP	Genotype			Allele Frequency (%)	
***MTHFR***	**CC**	**CT**	**TT**	**C**	**T**
C677T (*n* = 565)	295 (52.2%)	218 (38.6%)	52 (9.2%)	71.5	28.5
***MTHFR***	**AA**	**AC**	**CC**	**A**	**C**
A1298C (*n* = 565)	301 (53.3%)	196 (34.7%)	68 (12.0%)	70.6	29.4
***TCNII***	**AA**	**AG**	**GG**	**A**	**G**
A67G (*n* = 301)	252 (83.7%)	43 (14.3%)	6 (2%)	90.9	9.1
***TCNII***	**CC**	**CG**	**GG**	**C**	**G**
C776G (*n* = 292)	83 (28.4%)	141 (48.3%)	68 (23.3%)	52.6	47.4
***MTR***	**AA**	**AG**	**GG**	**A**	**G**
A2756G (*n* = 218)	128 (58.7%)	82 (37.6%)	8 (3.7%)	77.5	22.5
***MTRR***	**AA**	**AG**	**GG**	**A**	**G**
A66G (*n* = 246)	75 (30.5%)	114 (46.3%)	57 (23.2%)	53.7	46.3

**Table 3:** Plasma concentrations of vitamins and metabolic markers (tHcy, Folate, Holo-TC, tCbl, MMA; mean ± SD) according to the *MTHFR*, *TCN II*, *MTR* and *MTRR* genotypes in the 9-yo group.

	9-Year-Old				
SNP	tHcy (µmol/L)	Folate (nmol/L)	Holo-TC (pmol/L)	tCbl (pmol/L)	MMA (µmol/L)
***MTHFR*** **C677T**					
CC	3.7 ± 1.9 (*n* = 115)	23.1 ± 9.0 (*n* = 109)	101.8 ± 30.2 (*n* = 108)	502.2 ± 156.3 (*n* = 110)	0.274 ± 0.300 (*n* = 107)
CT	3.7 ± 1.7 (*n* = 65)	23.4 ± 9.7 (*n* = 60)	100.0 ± 33.7 (*n* = 59)	527.6 ± 178.6 (*n* = 63)	0.270 ± 0.198 (*n* = 62)
TT	4.5 ± 1.8 (*n* = 15)	17.4 ± 7.1 (*n* = 14)	92.7 ± 35.41 (*n* = 12)	555.3 ± 176.2 (*n* = 14)	0.295 ± 0.210 (*n* = 15)
***MTHFR*** **A1298C**					
AA	3.7 ± 1.8 (*n* = 101)	23.4 ± 10.1 (*n* = 97)	101.3 ± 33.7 (*n* = 93)	511.1 ± 157.8 (*n* = 98)	0.284 ± 0.252 (*n* = 97)
AC	3.8 ± 1.8 (*n* = 59)	22.1 ± 8.4 (*n* = 52)	98.2 ± 27.2 (*n* = 53)	544.5 ± 186.9 (*n* = 57)	0.225 ± 0.599 (*n* = 56)
CC	3.8 ± 2.0 (*n* = 35)	22.1 ± 7.8 (*n* = 34)	102.6 ± 33.0 (*n* = 33)	472.9 ± 141.2 (*n* = 32)	0.334 ± 0.448 (*n* = 31)
***TCN2*** **A67G**					
AA	3.9 ± 1.8 (*n* = 146)	22.8 ± 9.5 (*n* = 137)	101.2 ± 29.9 (*n* = 117)	523.0 ± 168.2 (*n* = 127)	0.277 ± 0.238 (*n* = 139)
AG	3.2 ± 2.0 (*n* = 37)	22.1 ± 7.9 (*n* = 35)	92.1 ± 26.4 (*n* = 33)	489.8 ± 178.8 (*n* = 35)	0.221 ± 0.054 (*n* = 35)
GG	3.7 ± 2.4 (*n* = 5)	26.1 ± 8.3 (*n* = 5)	87.9 ± 40.6 (*n* = 3)	561.2 ± 100.3 (*n* = 3)	0.153 ± 0.043 (*n* = 4)
***TCN2*** **C776G**					
CC	4.0 ± 1.8 (*n* = 59)	22.5 ± 8.7 (*n* = 54)	96.8 ± 29.2 (*n* = 48)	496.8 ± 171.8 (*n* = 56)	0.241 ± 0.063 (*n* = 55)
CG	3.6 ± 1.9 (*n* = 74)	21.5 ± 9.4 (*n* = 70)	101.2 ± 29.8 (*n* = 64)	525.1 ± 160.7 (*n* = 69)	0.281 ± 0.306 (*n* = 70)
GG	3.8 ± 2.0 (*n* = 42)	23.8 ± 8.9 (*n* = 40)	98.0 ± 29.7 (*n* = 61)	529. 8 ± 182.1 (*n* = 40)	0.287 ± 0.267 (*n* = 40)
***MTR*** **A2756G**					
AA	3.9 ± 1.5 (*n* = 70)	23.8 ± 10.2 (*n* = 64)	102.2 ± 25.4 (*n* = 63)	538.9 ± 188.8 (*n* = 68)	0.251 ± 0.084 (*n* = 67)
AG	3.7 ± 1.7 (*n* = 44)	23.6 ± 9.4 (*n* = 43)	99.0 ± 33.7 (*n* = 44)	531.0 ± 152.8 (*n* = 39)	0.325 ± 0.403 (*n* = 42)
GG	4.8 ± 3.3 (*n* = 2)	21.7 ± 7.6 (*n* = 2)	87.4 ± 28.2 (*n* = 2)	467.7 ± 33.4 (*n* = 2)	0.234 ± 0.044 (*n* = 2)
***MTRR*** **A66G**					
AA	3.9 ± 1.8 (*n* = 36)	24.8 ± 9.8 (*n* = 33)	109.0 ± 32.0 (*n* = 35)	530.8 ± 180.8 (*n* = 34)	0.257 ± 0.159 (*n* = 35)
AG	3.8 ± 1.6 (*n* = 60)	21.8 ± 9.1 (*n* = 55)	100.4 ± 34.7 (*n* = 55)	537.0 ± 165.7 (*n* = 56)	0.262 ± 0.242 (*n* = 56)
GG	4.0 ± 1.8 (*n* = 30)	22.9 ± 9.1 (*n* = 27)	100.4 ± 25.8 (*n* = 26)	513.8 ± 183.8 (*n* = 28)	0.354 ± 0.461 (*n* = 29)

*p* values for differences between means of the three genotypes were tested with one-way analysis of variance (ANOVA). *p* values < 0.05 were considered statistically significant. No statistically significant differences were observed for any of the evaluated biomarkers among the different genotypes. Total Homocysteine (tHcy); total cobalamin (tCbl); holo-transcobalamin (Holo-TC); methylmalonic acid (MMA).

**Table 4:** Plasma concentrations of vitamins and metabolic markers (tHcy, Folate, Holo-TC, tCbl, MMA; mean ± SD) according to the *MTHFR*, *TCN II*, *MTR* and *MTRR* genotypes in the 17-yo group.

	17-Year-Old				
SNP	tHcy (µmol/L)	Folate (nmol/L)	Holo-TC (pmol/L)	tCbl (pmol/L)	MMA (µmol/L)
***MTHFR* C677T**					
CC	**8.4** **± 2.4 (*n* = 62)** *	11.9 ± 3.4 (*n* = 34)	71.9 ± 26.3 (*n* = 33)	307.6 ± 114.2 (*n* = 32)	0.170 ± 0.101 (*n* = 55)
CT	**8.8** **± 3.9 (*n* = 48)** *	11.8 ± 5.1 (*n* = 34)	63.8 ± 24.3 (*n* = 34)	305.4 ± 124.2 (*n* = 32)	0.175 ± 0.092 (*n* = 39)
TT	**11.3 ± 5.0 (*n* = 12)** *	9.0 ± 9.2 (*n* = 7)	51.4 ± 19.1 (*n* = 7)	250.6 ± 124.4 (*n* = 6)	0.252 ± 0.161 (*n* = 10)
***MTHFR* A1298C**					
AA	9.4 ± 3.0 (*n* = 58)	11.6 ± 4.3 (*n* = 35)	69.5 ± 27.9 (*n* = 35)	320.4 ± 131.6 (*n* = 33)	0.190 ± 0.114 (*n* = 47)
AC	8.5 ± 4.0 (*n* = 46)	11.2 ± 3.7 (*n* = 25)	63.2 ± 19.1 (*n* = 25)	292.0 ± 100.7 (*n* = 23)	0.162 ± 0.067 (*n* = 41)
CC	7.9 ± 3.1 (*n* = 18)	12.2 ± 4.9 (*n* = 15)	63.7 ± 28.5 (*n* = 14)	273.5 ± 115.9 (*n* = 14)	0.197 ± 0.155 (*n* = 16)
***TCN2* A67G**					
AA	8.7 ± 3.5 (*n* = 105)	12.0 ± 4.4 (*n* = 62)	66.2 ± 25.5 (*n* = 61)	298.8 ± 119.6 (*n* = 58)	0.183 ± 0.113 (*n* = 90)
AG	10.3 ± 2.5 (*n* = 6)	9.6 ± 2.6 (*n* = 4)	68.1 ± 30.1 (*n* = 4)	286.1 ± 71.5 (*n* = 4)	0.146 ± 0.010 (*n* = 5)
GG	(*n* = 1)	-	-	-	(*n* = 1)
***TCN2* C776G**					
CC	8.9 ± 2.4 (*n* = 24)	9.6 ± 4.5 (*n* = 13)	73.2 ± 28.8 (*n* = 13)	292.0 ± 107.8 (*n* = 13)	0.154 ± 0.073 (*n* = 21)
CG	8.8 ± 3.2 (*n* = 66)	12.5 ± 4.5 (*n* = 44)	65.8 ± 25.1 (*n* = 44)	314.5 ± 125.2 (*n* = 41)	0.194 ± 0.128 (*n* = 55)
GG	8.9 ± 5.0 (*n* = 26)	10.4 ± 3.5 (*n* = 14)	63.7 ± 24.5 (*n* = 13)	279.8 ± 131.0 (*n* = 12)	0.175 ± 0.074 (*n* = 23)
***MTR* A2756G**					
AA	8.6 ± 2.4 (*n* = 57)	11.9 ± 4.5 (*n* = 35)	68.7 ± 28.8 (*n* = 34)	**333.0 ± 126.3 (*n* = 31)** *	0.188 ± 0.124 (*n* = 50)
AG	8.6 ± 2.7 (*n* = 38)	11.6 ± 4.2 (*n* = 22)	67.1 ± 20.8 (*n* = 22)	**283.1 ± 96.9 (*n* = 22)** *	0.165 ± 0.062 (*n* = 31)
GG	10.1 ± 7.3 (*n* = 6)	10.5 ± 3.0 (*n* = 5)	61.1 ± 31.8 (*n* = 5)	**200.2 ± 112.3 (*n* = 5)** *	0.230 ± 0.193 (*n* = 6)
***MTRR* A66G**					
AA	8.8 ± 3.8 (*n* = 38)	11.3 ± 3.9 (*n* = 24)	64.3 ± 27.8 (*n* = 24)	337.4 ± 138.6 (*n* = 22)	0.193 ± 0.141 (*n* = 33)
AG	8.6 ± 2.5 (*n* = 54)	12.0 ± 4.7 (*n* = 33)	68.9 ± 27.5 (*n* = 32)	282.8 ± 110.2 (*n* = 31)	0.178 ± 0.076 (*n* = 48)
GG	8.8 ± 2.4 (*n* = 27)	11.4 ± 3.8 (*n* = 16)	65.6 ± 17.3 (*n* = 16)	297.4 ± 106.2 (*n* = 15)	0.165 ± 0.107 (*n* = 22)

* *p* value statistically significant. *p* values for differences between means of the three genotypes were tested with one-way analysis of variance (ANOVA). *p* values < 0.05 were considered statistically significant. Significantly different plasma concentrations were found for tHcy between *MTHFR* 677 three genotypes (*p* = 0.027) and for tCbl between *MTR 2756* genotypes (*p* = 0.041). Total Homocysteine (tHcy); total cobalamin (tCbl); holo-transcobalamin (Holo-TC); methylmalonic acid (MMA).
